# A Trust Wide Service Evaluation to Explore the Reasons for Discontinuation of Clozapine and Its impact.

**DOI:** 10.1192/bjo.2026.11626

**Published:** 2026-06-30

**Authors:** Aysha Zabin Madathil, Sumeet Gupta, Nur Liyana Muhamad, Olufunto Segun, Matthew Croft

**Affiliations:** 1 Tees Esk and Wear Valleys NHS Foundation Trust, Harrogate, United Kingdom; 2 Tees, Esk and Wear Valleys NHS Foundation Trust, Harrogate, United Kingdom; 3 Leeds and York Partnership NHS Foundation Trust, Leeds, United Kingdom; 4 Bradford District Care NHS foundation Trust, Leeds, United Kingdom

## Abstract

**Aims:**

Clozapine is the recommended medication for patients with treatment resistant schizophrenia and uniquely effective with the absence of extrapyramidal side effects. However, clozapine discontinuation is common in clinical practice with clinicians resorting to treatments with supported by lower quality evidence.

We aimed to identify the reasons for discontinuation of clozapine retrospectively for a duration of 30 years in a large mental health trust. We also intend to develop guidance and training for clinicians to ensure the safe and effective use of clozapine as underutilisation and delay in commencement are a priority both locally and internationally.

**Methods:**

Using the Clozapine Patient Monitoring System (CPMS), between 1994 and 2024, patients who discontinued clozapine due to any reasons were identified. Electronic patient records were then reviewed to detail the reasons and whether it was decided by the clinician or the patient. We also noted the timeline of stopping clozapine since the initiation.

Data collected also included demographic characteristics, mental health diagnoses, comorbidities, additional medications, side effects noted while on clozapine, date of starting and ending clozapine and strategies for managing the side effects. For those patients with neutropenia, additional data were gathered separately.

**Results:**

A total of 1184 patients were started on clozapine during the study period. Out of these, 225( 19%) patients discontinued at various timeline in the course of treatment up to 31 August 2024 and their data were analysed.

The number of patients discontinued were mostly during the early 3 months( 38.2%) and after one year( 35.1%).Adverse effects account for 72% (n=62), 62% (n=37) and 48% (n=38) of discontinuations at <3months, 3-12 months and >12 months respectively suggesting those are the primary cause of discontinuation up to 12 months with other factors becoming equally important beyond 12 months.

Clinicians are consistently more likely to discontinue clozapine than patients (61% of total discontinuation)

Twenty-eight patients stopped due to red results. Interestingly, neutropenia accounts for more discontinuation than sedation.

**Conclusion:**

The percentage of patients discontinued clozapine were less compared to previous research evidence. Most patients could potentially have continued clozapine, by proactive management of the adverse effects and better psychoeducation.

We will be recommending education and training events for all clinicians involved in clozapine management to discuss further strategies so that clozapine can be utilised promptly by the clinicians with confidence.

